# Dietary Intervention and DEHP Reduction

**DOI:** 10.1289/ehp.1103852

**Published:** 2011-09-01

**Authors:** Stephen P. Risotto

**Affiliations:** American Chemistry Council, Washington, DC, E-mail: steve_risotto@americanchemistry.com

Rudel et al. (2011) reported a surprising reduction in metabolites of bis(2-ethylhexyl) phthalate (DEHP) in their dietary intervention study, considering that—to the best of the industry’s knowledge—the plasticizer is no longer used in the food packaging products that the authors removed from the subjects’ dietary routine. Although we question the public health significance of a potential reduction of a few micrograms per liter of DEHP metabolites, we initially saw the study as having the potential to improve our understanding of how low-level exposure to DEHP, suggested by the presence of the metabolites, may be occurring. Unfortunately, in reviewing the Rudel et al. analysis more thoroughly, we were disappointed.

The 56% reduction in mean levels suggested by Rudel et al. (2011) is based on the concentration of DEHP metabolites—before correcting for creatinine levels. With little more than a sentence, Rudel et al. dismissed the accepted practice of correcting for creatinine levels to account for the substantial variability in an individual’s urine output. They suggested that such adjustment may “bias associations between urine metabolite concentrations and age or sex” (Rudel et al. 2011) without explaining that the correction is widely used in urinary biomonitoring (by the Centers for Disease Control and most others) to improve the comparability of measurements across individuals.

To their credit, Rudel et al. (2011) did conduct a comparison of the creatinine-adjusted levels of DEHP metabolites and found no statistically significant difference in the mean levels of two of the three metabolites before and after dietary intervention. The authors did not report the change in the adjusted levels of the third metabolite in the article.

The authors also did not address the variability in preintervention levels among the study participants. The presence of two individuals with very high metabolite levels clearly skewed the mean value upward and, consequently, exaggerated the significance of the intervention. Although Table 2 of Rudel et al. (2011) provides the minimum, mean, and maximum values, the variability is best seen in their Supplemental Material, Figure 3 (doi:10.1289/ehp.1003170), and on Silent Spring Institute’s web site (Silent Spring Institute 2011). It is unfortunate that Rudel et al. (2011) chose not to address the variability in their article—and a bit surprising— because the postintervention increase in DEHP metabolites was significantly lower than the reported decrease (16% versus 56%).
